# Fatigue as a manifestation of psychosocial distress in a low‐income country: a population‐based panel study

**DOI:** 10.1111/tmi.12658

**Published:** 2016-01-25

**Authors:** Caroline Smartt, Girmay Medhin, Atalay Alem, Vikram Patel, Michael Dewey, Martin Prince, Charlotte Hanlon

**Affiliations:** ^1^ Centre for Global Mental HealthInstitute of Psychiatry, Psychology and NeuroscienceKing's College LondonLondonUK; ^2^ Aklilu‐Lemma Institute of PathobiologyAddis Ababa UniversityAddis AbabaEthiopia; ^3^ Department of PsychiatryAddis Ababa UniversityAddis AbabaEthiopia; ^4^ Centre for Global Mental HealthLondon School of Hygiene and Tropical MedicineLondonUK; ^5^ Centre for Chronic Conditions and InjuriesPublic Health Foundation of IndiaSangathGoaIndia; ^6^ Health Services and Population Research DepartmentInstitute of Psychiatry, Psychology and NeuroscienceKing's College LondonLondonUK

**Keywords:** fatigue, low‐and middle‐income countries, psychosocial distress, maternal CMD, Ethiopia, population‐based panel study, fatigue, PFR‐PRI, détresse psychosociale, maternal CMD, Ethiopie, étude de panel basée sur la population

## Abstract

**Objective:**

Fatigue is a common complaint worldwide and associated with disability and high health service use costs. We tested the hypothesis that maternal fatigue would be associated independently with maternal common mental disorder (‘maternal CMD’) in a rural, low‐income country setting.

**Methods:**

The analysis was conducted using data from a population‐based cohort located in the Butajira demographic surveillance site, Ethiopia. A total of 1065 women were recruited in pregnancy and followed up to 2.5 (*n* = 1009; 94.7%) and 3.5 years post‐partum (*n* = 989; 92.9%). Maternal CMD symptoms were measured using a locally validated version of the Self‐Reporting Questionnaire and fatigue was measured using a dichotomised item from the Patient Health Questionnaire‐15. Physical health indicators included haemoglobin level, body mass index and illness episodes. Generalised estimating equations were used to conduct hypothesis‐driven and exploratory multivariable analyses in the panel at 2.5 and 3.5 years.

**Results:**

The prevalence of maternal fatigue was 8.3% at 2.5 years and 5.5% at 3.5 years post‐partum. Psychological symptoms of maternal CMD were associated independently with complaints of fatigue after adjusting for anaemia, body mass index, physical ill health, poverty and other confounding variables: adjusted odds ratio (aOR), 1.46; 95% confidence interval (CI), 1.28–1.66 for each one point increase in SRQ score. In the multivariable model, only psychosocial factors (CMD and stressful life events) and self‐reported physical ill health were associated significantly with complaints of fatigue.

**Conclusion:**

Complaints of fatigue are associated strongly with maternal CMD and other psychosocial risk factors in this rural, low‐income country setting with a high burden of undernutrition and infectious disease. Fatigue should be understood as a potential indicator of CMD in primary care to improve detection and treatment.

## Abstract (fr)

**Objectif:**

La fatigue est une plainte fréquente dans le monde entier, associée à l'invalidité et à l'utilisation de dépenses élevées des services de santé. Nous avons testé l'hypothèse que la fatigue maternelle serait associée de façon indépendante au trouble mental courant maternel («CMD maternel») dans un cadre rural d'un pays à faible revenu.

**Méthodes:**

L'analyse a été réalisée en utilisant les données d'une cohorte basée sur la population, située dans le site de surveillance démographique de Butajira, en Ethiopie. 1065 femmes ont été recrutées durant la grossesse et suivies jusqu’à 2,5 (n = 1009; 94,7%) et 3,5 années (n = 989; 92,9%) post‐partum. Les symptômes de CMD maternel ont été mesurés en utilisant une version validée localement du Questionnaire d'Auto‐Déclaration et la fatigue a été mesurée en utilisant un élément dichotomique du Questionnaire de Santé du Patient #15. Les indicateurs physiques de santé comprenaient le taux d'hémoglobine, l'indice de masse corporelle (IMC) et les épisodes de maladie. Des équations d'estimation généralisée ont été utilisées pour mener des analyses basées sur l'hypothèse et des analyses multivariées exploratoires dans le panel à 2,5 et à 3,5 ans.

**Résultats:**

La prévalence de la fatigue maternelle était de 8,3% à 2,5 ans et de 5,5% à 3,5 ans post‐partum. Les symptômes psychologiques de CMD maternel étaient indépendamment associés à des plaintes de fatigue après ajustement pour l'anémie, l’IMC, la mauvaise santé physique, la pauvreté et d'autres facteurs confusionnels: odds ratio ajusté (AOR): 1,46; d'intervalle de confiance (IC) 95%: 1,28 à 1,66 pour chaque augmentation d'un point du score SRQ. Dans le modèle multivarié, seuls les facteurs psychosociaux (CMD et événements stressants de la vie) et la mauvaise santé physique auto‐déclarée étaient associés de façon significative avec les plaintes de fatigue.

**Conclusion:**

Les plaintes de fatigue sont fortement associées au CMD maternel et d'autres facteurs de risque psychosocial dans ce cadre rural d'un pays à faible revenu avec une forte charge de malnutrition et de maladies infectieuses. La fatigue devrait être comprise comme un indicateur potentiel de CMD dans les soins primaires afin d'améliorer la détection et le traitement.

## Abstract (es)

**Objetivo:**

La fatiga es una queja común a nivel mundial y está asociada con discapacidad y un alto coste del uso de servicios sanitarios. Hemos evaluado la hipótesis de que la fatiga materna estaría asociada de forma independiente con un desorden mental común de la madre (‘DMC materno’) en una zona rural de un país con bajos ingresos.

**Métodos:**

Se realizó un análisis utilizando datos de una cohorte poblacional localizada en la zona bajo vigilancia demográfica de Butajira, en Etiopía. Se reclutaron 1065 mujeres embarazadas y se siguieron hasta 2.5 (n=1009; 94.7%) y 3.5 años después del parto (n=989; 92.9%). Se midieron los síntomas DMC materno utilizando una versión validada a nivel local del Cuestionario de Autoinforme (CAI) y se midió la fatiga utilizando un elemento dicotomizado del cuestionario PHQ – 15. Los indicadores de salud física incluían el nivel de hemoglobina, el índice de masa corporal y episodios de enfermedad. Se utilizaron ecuaciones de estimación generalizadas para conducir análisis multivariables guiadas por hipótesis y análisis multivariables exploratorios, en el panel, a los 2.5 y 3.5 años.

**Resultados:**

La prevalencia de fatiga materna era del 8.3% a los 2.5 años y del 5.5% a los 3.5 años postparto. Los síntomas psicológicos de DMC materno estaban asociados de forma independiente con quejas de fatiga después de ajustar para anemia, índice de masa corporal, mala salud física, pobreza y otras variables de confusión: proporción de variables ajustada (aOR) 1.46; intervalo de confianza (IC) 95% 1.28‐1.66 para cada aumento de un punto en el puntaje del CAI. En un modelo multivariable, solo los factores psicosociales (DMC y eventos de vida estresantes) y mala salud física auto‐reportada, estaban asociados de forma significativa con quejas de fatiga.

**Conclusión:**

Las quejas de fatiga están fuertemente asociadas con DMC materno y otros factores de riesgo psicosociales en este lugar rural, en un país con bajos ingresos, y con una alta carga de desnutrición y enfermedad infecciosa. La fatiga debería entenderse como un indicador potencial de DMC en atención primaria con el fin de mejorar la detección y el tratamiento.

## Introduction

The complaint of fatigue is a common problem worldwide, with prevalence ranging from 10 to 33% in population samples 1, 2. Fatigue can arise from a range of physical pathologies, physiological states or psychiatric disorders, as a somatic expression of emotional distress or as a combination of some or all of these causes 1, 3. In epidemiological studies from high‐income countries, a consistent association is seen between fatigue and psychiatric morbidity, disability and increased healthcare utilisation 4. Around 17% of people who present to health services with the symptom of fatigue meet criteria for current major depression, with 45% having experienced a lifetime common mental disorders, specifically depression and anxiety disorders 5. In low‐ and middle‐income countries (LMICs), however, fatigue is often assumed to be the result of nutritional deficiencies or infectious disease 2.

Somatic expression of emotional distress ‘somatisation’, for example as the symptom of fatigue, is reported to be the single most common reason worldwide for psychiatric problems to go undiagnosed in general health care 6. Misdiagnosis of somatised fatigue results in unnecessary healthcare expenditures and potential iatrogenic harm 7 as a consequence of unnecessary investigations and prescription of non‐indicated medications 2, and the failure to address the underlying emotional distress may exacerbate the problem and perpetuate a cycle of suffering 8.

Although the vast majority of research has been conducted in Western contexts, a cross‐sectional study of chronic fatigue in a population sample of women from Goa, India, found that the strongest associations were with psychological and psychosocial risk factors, not nutritional or physical health indicators 2. However, this finding was from a relatively well‐resourced peri‐urban setting in a middle‐income country and may not be generalisable to rural settings in low‐income countries. In more resource‐constrained settings, there is a heightened danger of category fallacy, whereby a Western diagnostic concept, such as somatisation, is misapplied to fatigue arising from common physical conditions, such as anaemia, malaria and diarrhoea, or from the experience of extreme poverty itself 9.

In this study, we examine the relationship between fatigue, maternal common mental disorders ‘maternal CMD’, other psychosocial factors, physical health and nutritional status in a population‐based panel study of mothers of young children in rural Ethiopia. Maternal CMD is prevalent in perinatal women in LMICs and in women with young children ‘maternal CMD’ and has been associated with adverse effects on maternal functioning and child nutrition, illness episodes, impaired development, poorer access to preventive health care and lower infant survival 10, 11, 12, 13, 14, 15. Our objective was to investigate the underlying explanation for a complaint of fatigue at repeated evaluations of the study women at 2.5 and 3.5 years post‐partum. We hypothesised that a complaint of fatigue would be a manifestation of maternal CMD and be independent of anaemia, nutritional level, physical health status and other potential confounders.

## Methods

### Study design and setting

This was a population‐based panel study. Data for this analysis were collected as part of the C‐MaMiE project (*C*hild health, growth and development in relation to *ma*ternal *me*ntal disorder *i*n *E*thiopia) conducted in the demographic surveillance site (DSS) in Butajira District, Gurage Zone, Southern Nations, Nationalities, and Peoples' Region (SNNPR) of Ethiopia. The Butajira DSS is located about 130 km south of the capital, Addis Ababa, and comprises ten administrative subdistricts, nine rural and one urban. Butajira town is at an altitude of 2131 metres. Full‐time enumerators visit all households in the DSS every 3 months to collect vital registration and other demographic data 16. The rural population relies heavily on smallholder mixed farming for its livelihoods and parts of the DSS are food insecure from a combination of overpopulation and intermittent drought. Fertility levels are high, with a total fertility rate of 4.9 births per woman 17. The majority of women receive no antenatal care (59.2%) or post‐natal care (93.2%) and give birth at home (93.5% of births in the last 5‐year period) 17.

### Study participants

A population‐based sample of 1065 women in the third trimester of pregnancy was recruited into the C‐MaMiE study between July 2005 and February 2006 18. Potential participants were identified by DSS enumerators during their routine house‐to‐house surveillance activities and then approached for participation by project data collectors. Eligible women were 15–49 years old, residing in the DSS and able to speak Amharic. All participating women provided informed consent. The C‐MaMiE data collectors were female high‐school graduates working for the project full time and trained to conduct interviews and anthropometric measurements by project principal investigators.

The C‐MaMiE study has had repeated contact with the participating women and children from 2005 to date. This analysis uses data from assessments at 2.5 and 3.5 years post‐partum (measured between 2008 and 2009), with each variable operationalised and measured using standardised methods at both time points. The timing of the measurements was determined by the primary objective of the C‐MaMiE cohort to examine the impact of maternal CMD on child development.

### Measures

Maternal fatigue was operationalised as a dichotomous variable using the item from the Patient Health Questionnaire (PHQ), 15‐item version 19, which asks: ‘In the last 1 month period, how bothered were you by feelings of fatigue?’. Responses of being ‘bothered a little’ or ‘bothered a lot’ by fatigue in the last 1 month were considered to be complaints of fatigue and responses of being ‘not bothered’ or experiencing ‘no fatigue’ were classified as ‘no symptoms of fatigue’.

Maternal CMD symptoms were measured as a continuous variable using a locally validated version of the Self‐Reporting Questionnaire, 20‐item version (SRQ‐20) 20. The SRQ contains several somatic items that are purported to indicate the presence of emotional disorder (items 1, 2, 3, 5, 7, 18, 19, 20, 21) 21, Exploratory factor analysis using maximum‐likelihood estimation and varimax rotation showed that the somatic items within the SRQ‐20 and the ‘psychological’ SRQ items loaded onto the same underlying construct in this sample. For the purpose of this study, therefore, these items were excluded to prevent any possibility of overlap with the dependent variable fatigue. The resulting 12‐item SRQ scale was composed of psychological manifestations of depression and anxiety ‘SRQpsy scale’. As the time points under consideration were distant in time from when the women had given birth to the index child, post‐natal depression was not included in the analysis.

**Figure 1:**
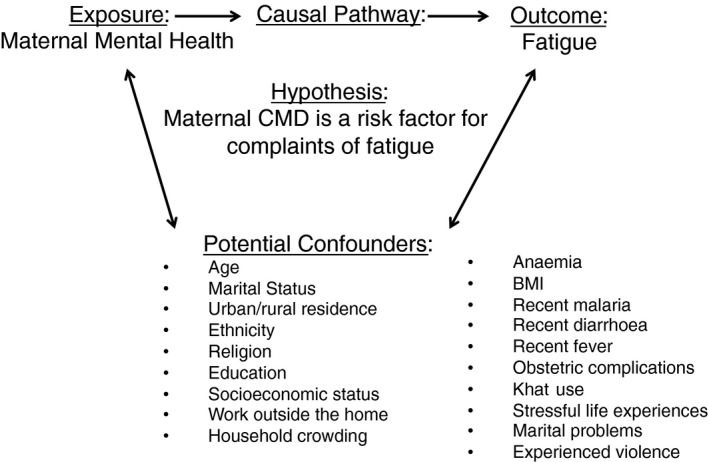
Conceptual framework linking maternal CMD and fatigue

Potential confounding variables were identified *a priori* according to the conceptual model presented in Figure 1 and were selected on the basis of their known associations with both fatigue and emotional distress.

Socio‐demographic and economic variables included age, marital status, urban/rural residence, ethnicity, religion, educational level, extent of the women's work outside the home and household crowding. A composite variable measuring socio‐economic status (SES) was created by adding the following poverty indicators: self‐reports of indebtedness, hunger in the last month and perceived lower relative wealth, and fulfilled criteria for a hierarchical Mokken scale 22.

#### Maternal health variables

The woman's pregnancy status was ascertained at each time point. Maternal self‐reported health states included malaria, diarrhoea and fever episodes (measured for the 1 month preceding each time point), obstetric complications in the index birth, and frequency of *khat* use. Both body mass index (BMI, calculated as weight in kg^2^/height in m) and haemoglobin (g/dl) were measured as continuous variables and dichotomised according to standard values for low BMI (<18.5 kg/m^2^) and moderate/severe anaemia, adjusted for altitude (≤101 g/l) 23. Maternal weight and height were measured using standard anthropometric techniques 18, and haemoglobin was measured with the portable, battery‐operated HemoCue analyser as previously utilised in the Ethiopian Demographic and Health Survey 17.

#### Psychosocial variables

The List of Threatening Experiences (LTE) was translated and adapted for the local context 24. The LTE covers twelve categories of life events associated with long‐term threat and was measured continuously based on total score. Problems in the marital relationship were measured as a composite variable on a 0–3 scale based on questions about frequency of quarrelling, obtaining inadequate help from the husband, and the overall quality of relationship being average, poor or very poor 22. Women were also asked about any experiences of violence in the last 1‐month period.

### Data analysis

Normally distributed continuous variables were summarised using mean with standard deviation (SD) as a measure of central tendency. For non‐normally distributed data, the median and 25 and 75th centiles were presented. For categorical variables, percentages were calculated to show how they were distributed in the sample.

### Hypothesis‐driven analysis

Generalised estimating equations (GEE) were used to determine population‐averaged associations from the correlated panel data collected at the two time points to investigate the association between maternal CMD and fatigue. Potential confounders were added in groups of variables (socio‐demographic, pregnancy status, socio‐economic, maternal health and behaviour, nutritional status and psychosocial variables) to the model including maternal CMD and fatigue to understand the impact of confounding on the association. A fully adjusted model then included all *a priori* confounders.

### Exploratory analysis

Crude odds ratios for the associations of the study variables with fatigue across the two time points were calculated using GEE. Variables associated with fatigue with a *P*‐value <0.20 were then retained in a multivariable model. Unmarried status and exposure to violence could not be included in the final multivariable model, however, due to small numbers.

## Results

At 2.5 years, 1009 (94.7%) women remained under follow‐up and 989 (92.9%) at 3.5 years. At both time points, women who were lost to follow up did not differ with respect to their CMD or fatigue status, educational level, religion or BMI; however, they were more likely to reside in an urban area and be younger.

### Descriptive characteristics

Table 1 presents the demographic, socio‐economic, health, nutrition and psychosocial characteristics of the study population at 2.5 and 3.5 years post‐partum. Mean maternal age at the time of the child's birth was 27.0 years (SD ± 6.4 years) in the women followed up at 2.5 years and 27.1 years (SD ± 6.4 years) and in the women followed up at 3.5 years. The largest ethnic group was Meskan (46.0%; *n* = 464), followed by Silti and Mareko. Over three quarters of the women were Muslim. Most women lived rurally and the vast majority were married. Only 20% had received any formal education. In total, 24.0% of women were pregnant at 2.5 years post‐partum and 14.7% at 3.5 years post‐partum. More women reported being bothered by fatigue at 2.5 years (8.3%, *N* = 84) than at 3.5 years (5.5% *N* = 54). Only 1% of women reported fatigue at both time points. The number of women scoring six or more on the SRQ‐20, and therefore meeting the study cut‐off for a diagnosis of CMD, was *N* = 55 (5. 5%) at 2.5 years and *N* = 48 (4.9%) at 3.5 years.

**Table 1:** Descriptive characteristics of the study women at the 2.5 year (*n* = 1009) and 3.5 year (*n* = 989) time points

Characteristic		Number at 2.5 years (%)	Number at 3.5 years (%)
Complaint of fatigue	Yes	84 (8.3)	54 (5.5)
Ethnicity	Meskan	464 (46.0)	455 (46.0)
	Mareko	139 (13.8)	139 (14.0)
	Silti	241 (23.9)	235 (23.8)
	Other	165 (16.3)	160 (16.2)
Religion	Muslim	784 (77.7)	770 (77.8)
	Orthodox Christian	151 (15.0)	146 (14.8)
	Protestant	62 (6.1)	61 (6.2)
	Catholic	12 (1.2)	12 (1.2)
Marital status	Married	975 (96.6)	950 (96.1)
Residence	Rural	880 (87.2)	866 (87.6)
Education	No formal education	807 (80.0)	791 (80.8)
Socio‐economic status^a^	0	507 (50.9)	537 (54.5)
	1	394 (39.5)	352 (35.7)
	2	76 (7.6)	67 (6.8)
	3	20 (2.0)	29 (2.9)
Days worked outside home	5 days or more/week	286 (31.5)	269 (30.6)
Pregnancy status	Currently pregnant	242 (24.0)	145 (14.7)
Obstetric complications	No obstetric complications	344 (35.5)	337 (35.4)
	1 obstetric complication	335 (34.6)	328 (34.5)
	≥2 obstetric complications	290 (29.9)	286 (30.1)
Maternal illness	Recent diarrhoea	154 (15.3)	95 (9.6)
	≥1 recent malaria episode	142 (14.1)	136 (13.8)
	Recent fever	301 (29.8)	241 (24.4)
Body mass index	<18.5 kg/m^2^	198 (20.0)	190 (19.4)
Plasma haemoglobin level^b^	Anaemia (≤101 g/l)	42 (8.0)	53 (10.1)
Maternal substance use	Chews khat weekly or more	305 (30.2)	317 (32.1)
Marital Relationship^c^	0	766 (80.2)	762 (81.7)
	1	127 (13.3)	117 (12.5)
	2	45 (4.7)	43 (4.6)
	3	17 (1.8)	11 (1.2)
Experience of violence	Experienced violence	15 (1.5)	18 (1.8)
Age (years)		Mean 27.0 (SD 6.4)	Mean 27.1 (SD 6.4)
Body mass index (kg/m^2^)		Mean 20.4 (SD 2.3)	Mean 20.5 (SD 2.4)
Common mental disorder symptoms (SRQpsy)		Median 0 (25 0, 75th 0)	Median 0 (25 0, 75th 0)
Number of stressful life events in past 6 months		Median 0 (25 0, 75th 1)	Median 0 (25 0, 75th 1)
Number of people in the household		Median 6 (25 4, 75th 7)	Median 6 (25 5, 75th 7)

SRQpsy, Self‐Reporting Questionnaire, psychological subscale.

a SES is the sum of three factors: indebtedness, experience of hunger in the last month and perceived lower wealth.

b Haemoglobin measured on a subsample of *n* = 526 and is adjusted for altitude.

c Marital relationship is a composite variable based on frequency of quarrelling, obtaining inadequate help from spouse and overall quality of the relationship being average, poor or very poor.

### Hypothesis‐driven analysis

Table 2 presents the results of the hypothesis‐driven analysis. Maternal CMD (SRQpsy score) was associated with increased odds of a complaint of fatigue in the GEE model: crude odds ratio (OR) = 1.64; 95% confidence interval = 1.49–1.80 for each one point increase in SRQ score. This association remained significant after adjusting for a wide range of potential confounding factors: adjusted odds ratio (aOR) = 1.46; 95% confidence interval (CI) = 1.28–1.66.

**Table 2:** Multivariable model for the association between psychological symptoms of maternal common mental disorder (CMD) and complaints of fatigue using correlated data at 2.5 and 3.5 years

Model	Odds Ratio for the association between CMD and fatigue (95% Confidence Interval)
Model 1: Unadjusted association between CMD and fatigue	1.64 (1.49–1.80)
Model 2: Model 1 + socio‐demographic factors *(age, urban/rural residence)*	1.62 (1.47–1.78)
Model 3: Model 2 + pregnancy status	1.63 (1.47–1.79)
Model 4: Model 3 + socio‐economic factors *(SES, education, crowding, women's outside work)*	1.59 (1.42–1.79)
Model 5a: Model 4 + self‐reported health status *(obstetric complications, diarrhoea, malaria, fever, khat use)*	1.52 (1.34–1.71)
Model 5b: Model 5a + nutritional status *(body mass index, anaemia)*	1.52 (1.34–1.71)
Model 6 (Fully Adjusted): Model 5b + psychosocial factors *(life events, problems in marital relationships)*	1.46 (1.28–1.66)

### Exploratory analysis

Table 3 presents the results of the GEE exploratory analysis. In univariate analysis, fatigue was associated significantly with psychological symptoms of maternal CMD, increasing age, low socio‐economic status, maternal ill health (diarrhoea, malaria and fever), stressful life events, marital problems and recent experience of violence. There was no association between maternal fatigue and having moderate–severe anaemia (crude OR 0.93; 95%CI 0.78—1.12) or with having a low BMI (crude OR 1.17; 95%CI 0.76—1.80). In the final multivariable model, maternal fatigue was associated significantly (*P* <0.05) with maternal CMD, fever and life events. Although we noted a marginally significant interaction between SRQpsy score and time, upon further examination, the interaction was found to be due to a few women outliers with higher SRQ scores.

**Table 3:** Exploratory multivariable model for factors associated with maternal fatigue in correlated data at the 2.5 and 3.5 year time points

Variable	Crude odds ratio (95% Confidence Interval; CI)	Adjusted odds ratio (95% CI)
Common mental disorder symptoms * (SRQ‐psychological subscale total score)*	1.64 (1.49–1.80)	1.51 (1.35–1.68)
Socio‐demographic characteristics		
Age (years)	1.05 (1.02–1.08)	1.02 (0.99–1.05)
Unmarried	0.19 (0.03–1.39)	–
Urban residence	0.83 (0.47–1.48)	–
Pregnant	1.29 (0.86–1.95)	–
Socio‐economic factors		
No formal education	0.85 (0.55–1.31)	–
Lower socio‐economic status	1.48 (1.20–1.83)	0.96 (0.74–1.26)
Crowded household	1.08 (0.99–1.18)	–
Works outside ≥5 days/week	1.28 (0.87–1.89)	–
Maternal health and behaviour		
Low body mass index (<18.5 kg/m^2^)	1.17 (0.76–1.80)	–
Anaemia (≤101 g/l)	0.93 (0.78–1.12)	–
Recent diarrhoea	2.94 (1.96–4.39)	1.58 (0.99–2.53)
Recent malaria	2.80 (1.89–4.15)	1.34 (0.84–2.14)
Recent fever	4.18 (2.93–5.98)	2.62 (1.71–3.99)
Obstetric complications		
None	(Reference)	–
1 obstetric complication	0.84 (0.54–1.33)	
≥2 obstetric complications	1.17 (0.76–1.81)	
Khat use	0.87 (0.59–1.28)	
Psychosocial Variables		
Recent life events	1.72 (1.50–1.98)	1.39 (1.16–1.67)
Marital problems	1.67 (1.35–2.08)	1.13 (0.87–1.47)
Recent experience of violence^a^	1.50 (1.26–1.79)	–
Time	0.96 (0.94–0.99)	0.97 (0.94–1.00)

a Recent experience of violence was excluded from the exploratory modelling due to the very small numbers.

## Discussion

In this population‐based panel study of women in rural Ethiopia, a consistent and strong association was found between maternal psychological symptoms of CMD and complaints of fatigue at repeated time points after adjusting for maternal anaemia, undernutrition, physical health, poverty and a range of other potential confounders. Maternal fatigue was also associated significantly with both recent fever and stressful life events, indicating that there is a psychosomatic component adding to the morbidity of fatigue in this population.

The women in this study reported relatively poor physical health and nutritional status, reflecting both the high infectious disease burden and prevailing poverty in this rural Ethiopian setting: 20% of women had a low BMI and between 8 and 10% had moderate or severe anaemia. In low‐income countries, fatigue is often assumed to arise from factors related to undernutrition or physical ill health 2. Our results support previous findings from a more advantaged population of women in a middle‐income country 2 and underscore the relevance of fatigue as an indicator of psychological and psychosocial distress in this rural Ethiopian setting.

Strengths of this analysis include the rural population base, the use of a pre‐validated measure for CMD and two objective measures of physical health status. We were able to make use of repeated measures in the same cohort to examine population‐averaged cross‐sectional associations between psychological symptoms of CMD and fatigue. A further strength of the C‐MaMiE cohort is the consistent, long‐standing relationship between the data collectors and study participants. Many topics in the C‐MaMiE questionnaire are sensitive, so familiarity and trust between the women in the study and the women working for it likely improves interview quality.

A limitation was the cross‐sectional nature of the analysis. It was therefore not possible to know whether CMD caused fatigue, fatigue caused CMD, or indeed whether fatigue is a manifestation of CMD and they are actually the part of the same phenomena. Because we ascertained outcome at the same time that we measured CMD, somatic symptoms and other psychosocial exposures, we cannot rule out the possibility that negative recall bias inflated the associations found between fatigue and psychosocial factors. However, recall bias would have no bearing on the lack of associations with the objective measures of BMI and haemoglobin, so our conclusions about their relative importance appear to hold true. Although the PHQ‐15 fatigue item had not been validated in the study area, care was taken to ensure semantic and conceptual validity during translation. Furthermore, concurrent validity was indicated through the association with disability scores (data available from authors on request).

A number of previous studies from sub‐Saharan Africa have explored the relationship between somatisation, CMD and other forms of psychological distress, with varied findings. An early study from Nigeria developed a somatic symptom scale comprised entirely of local somatic metaphors. When administered to people in contact with mental health care, women were found to reported somatic symptoms more frequently than men. The authors concluded that expression of psychological disturbance in form of somatic metaphor is culturally acceptable for women, who otherwise are not permitted to be psychiatrically ill 25. Another Nigerian study described the creation of a Nigerian version of the SRQ‐20 constructed by adding culturally specific symptoms to the original twenty SRQ items 26. A third Nigerian survey found that core depressive symptoms were better predictors of depression than the somatic symptoms they studied and that somatic and psychological symptoms grouped separately in factor analysis. Based on their findings, they argued that depression can be diagnosed with certainty only if specific core depressive symptoms are enquired about and that somatic symptoms should be secondary in the diagnostic process 27. In Kenya, however, a culturally specific questionnaire showed high agreement to multiple international scales measuring depression, with the highest correlation found to self‐rating scales measuring somatisation 28. Although important for enriching the somatisation literature and elucidating some of the mechanisms by which somatisation and CMD are related in African contexts, these studies are all limited by their use of highly educated, urban samples drawn largely from major psychiatric and general hospital settings as well as the lack of assessment for underlying physical pathology, in contrast to the methods employed in this analysis.

The prevalence of fatigue in this population was lower than has been found elsewhere 1, 2. The lower values seen in this population may reflect cultural differences in terms of how fatigue is conceptualised and described in oral interviews outside of a conventional medical setting. It is clear that somatic presentations in primary healthcare settings are extremely common in sub‐Saharan Africa, as they are worldwide; however, the frequency of specific somatic complaints and idioms varies culturally 29. Overall, fatigue as somatised distress appears to be widespread and this study demonstrates its importance in Ethiopia.

Our findings have implications for current treatment approaches to the management of fatigue in similar rural settings in LMICs, which most often do not consider the mental health status of the patient. In Tanzania, fatigue was found to be a very common presenting complaint in both biomedical and traditional healer clinics 30; however, practitioners in LMICs seldom have appropriate algorithms for determining the aetiology and appropriate treatment for complaints of fatigue. In India, fatigue is reported to be frequently mismanaged with prescriptions of non‐indicated tonics, vitamin and analgesic injections, and benzodiazepine medications, all of which complicate and potentially worsen patients' health 2. There is a pressing need for further research to improve evidence‐based guidelines for the management of fatigue in LMIC treatment settings.

In common with many other LMICs, Ethiopia is in the process of implementing the World Health Organization's mental health Gap Action Programme 31 which seeks to scale‐up mental health care through integration into primary care settings 32. Detection and management of depression in primary care settings is prioritised for action within mhGAP. Our study, in combination with the previous evidence from India, now provides a strong basis to inform primary healthcare workers of the relevance of mental health to commonly encountered presentations of somatic symptoms, in particular fatigue. Such a message is vital for changing primary care worker attitudes to mental health and improving detection of depression, both of which underpin the success of mhGAP.

Further research in LMICs needs to examine the social and economic consequences of somatic manifestations of CMD and the impact on health systems. This will help in reprioritising constrained resources and stimulating efforts to provide effective and necessary treatments. There is evidence suggesting that multiple somatic complaints in the absence of comorbid CMD may require distinct interventions 33. There is therefore a great need for more randomised controlled trials in LMICs to evaluate various treatments and interventions found to be successful elsewhere 34.

This study contributes to the epidemiology of fatigue in this rural, low‐income country setting and shows it is closely associated with psychological and psychosocial factors among rural women in a low‐income country. Of the physical health indicators, only report of a recent episode of fever was associated with reports of fatigue, and there was no association with reported diarrhoea or malaria or with objective measures of BMI and haemoglobin. Therefore, although physical health causes need to be excluded by primary care workers, the predominant associations with fatigue were psychosocial. To improve detection and treatment of CMD in primary care in LMICs, the WHO's mhGAP management algorithms for CMDs need to give greater emphasis to somatic symptoms, such as fatigue, as a common presenting complaint of CMD.
